# Furnishing Wound Repair by the Subcutaneous Fascia

**DOI:** 10.3390/ijms22169006

**Published:** 2021-08-20

**Authors:** Dongsheng Jiang, Yuval Rinkevich

**Affiliations:** 1 Institute of Lung Biology and Disease, Helmholtz Zentrum München, Max-Lebsche-Platz 31, 81377 Munich, Germany; dongsheng.jiang@helmholtz-muenchen.de; 2 Institute of Regenerative Biology and Medicine, Helmholtz Zentrum München, Max-Lebsche-Platz 31, 81377 Munich, Germany

**Keywords:** fascia, subcutaneous fascia, superficial fascia, skin, wound healing, fibrosis, scar

## Abstract

Mammals rapidly heal wounds through fibrous connective tissue build up and tissue contraction. Recent findings from mouse attribute wound healing to physical mobilization of a fibroelastic connective tissue layer that resides beneath the skin, termed subcutaneous fascia or superficial fascia, into sites of injury. Fascial mobilization assembles diverse cell types and matrix components needed for rapid wound repair. These observations suggest that the factors directly affecting fascial mobility are responsible for chronic skin wounds and excessive skin scarring. In this review, we discuss the link between the fascia’s unique tissue anatomy, composition, biomechanical, and rheologic properties to its ability to mobilize its tissue assemblage. Fascia is thus at the forefront of tissue pathology and a better understanding of how it is mobilized may crystallize our view of wound healing alterations during aging, diabetes, and fibrous disease and create novel therapeutic strategies for wound repair.

## 1. Introduction

The Fascia Research Society [1] defines fascia anatomically as: “a sheath, a sheet, or any other dissectible aggregations of connective tissue that forms beneath the skin to attach, enclose, and separate muscles and other internal organs”. Fascia is an uninterrupted viscoelastic connective tissue (fibroelastic) that extends and interconnects between tissues and organs throughout the body [2]. It bridges muscles, bones, nerves, and internal organs, in a unifying system of connective tissues termed broadly as the “fascial system” [3,4]. A broader terminology of the “fascial system” therefore extends to all fibroelastic soft, collagen containing, loose and dense fibrous connective tissues that permeate the body. It includes elements such as superficial and deep fascia, epineurium, joint capsules, meninges, myofascial expansions, periostea, retinacula, septa, visceral fascia, adventitia and neurovascular sheaths, aponeuroses, and all the intramuscular and intermuscular connective tissues including endo-, peri-, and epimysium [1].

As one of the primary connective tissues of the mammalian body plan, fascia originates during embryogenesis from the embryonic mesoderm [5]. However, the exact mesodermal origins of the fascial system are unclear, as is the question of whether it has single or multiple origins. This is an important distinction because rather than a single homogeneous connective tissue layer, the fascial system encompasses various layers at different depths, forming a mechanical matrix that stabilizes and maintains tissue and organ strength and pliancy. Fascia equally regulates extracellular fluid flow [6,7]. The fascial system uniquely acts as a physically compliant interface between adjacent more dense connective tissue layers due to its areolar structure and viscous properties and low dynamic modulus. Cadaveric and animal studies and in vivo trials suggest that these attributes enable fascia to serve as a primary mechanotransduction system of the body’s soft tissues.

## 2. Anatomy of Subcutaneous Fascia

Three major types of fasciae are described, based on tissue location: (1) the subcutaneous fascia that is present underneath the skin, (2) the visceral fascia that envelopes internal organs, and (3) the muscle fascia that envelops and interconnects between muscle fibers.

The subcutaneous fascia, also known as superficial fascia, is composed of a single or several layers of loose areolar connective tissues, depending on the anatomic skin location. The different layers of the subcutaneous fascia are further interconnected to the skin and to the deep fascia by perpendicularly oriented fibrous septa called retinacula [8]. The fascial tissues that extend from the superficial fascia toward the hypodermis and dermis are termed as retinaculum cutis superficialis, and those fascial tissues that extend toward more deep fascia are termed as retinaculum cutis profundus [9]. The subcutaneous fascia and its associated retinacula form a three-dimensional network that mechanically link the skin, subcutaneous layer, and deeper muscle layer, to provide a dynamic anchor to the skin [10,11,12]. Subcutaneous fascia is an elastic layer of connective tissue, formed by loosely packed interwoven collagen fibers mixed with abundant elastic fibers [6,8], making it a unique fibroelastic layer that is easily stretched in various directions and then returned to its initial state.

The subcutaneous fascia plays a critical role in maintaining skin integrity and supporting and sustaining subcutaneous structures including, but not limited to, blood vessels, lymphatic vessels, nerve bundles, and adipose tissue [13,14]. Thick retinacula cutis divide fat into little lobules and anchor the skin to the superficial fascia [8]. Fascia also facilitates the sliding of the dermal layers over the underlying muscle and bone [5].

**Figure 1:**
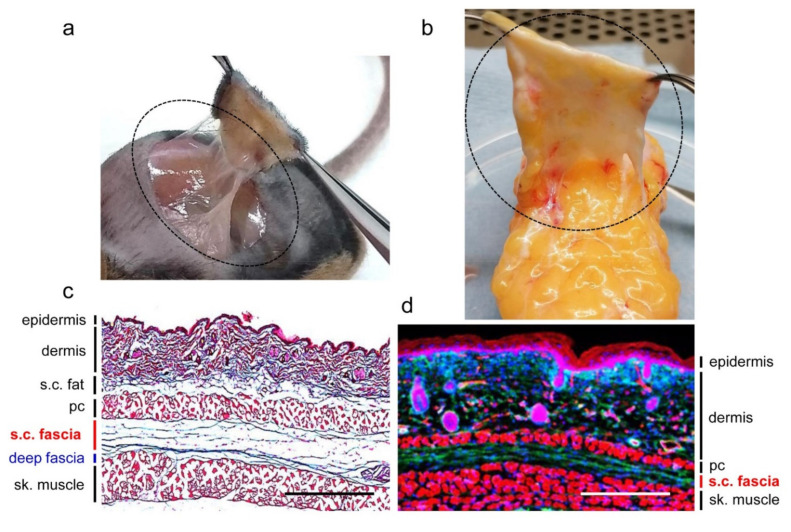
Subcutaneous fascia of mouse and human. (**a**) Macroscopic photo of mouse back skin subcutaneous fascia. (**b**) Macroscopic photo of human back skin subcutaneous fascia. The dotted circles indicate the subcutaneous fascia of mouse and human. (**c**), Masson’s trichrome staining of back skin of an adult mouse including all skin layers, subcutaneous fascia, and skeletal muscle wrapped with deep fascia. Collagen fibers are stained in blue. (**d**) Fluorescence microscopic image of back skin from a fibroblast lineage specific transgenic mouse line (*En1*^Cre^; *R26*^mTmG^) showing subcutaneous fascia is enriched with En1 lineage positive scar-forming fibroblasts (GFP^+^). pc, panniculus carnosus; s.c., subcutaneous; sk., skeletal. Scale bars: 500 µm. The images are derived from our work and are not published elsewhere before.

In loose-skinned mammals such as rodents, cats, dogs, horses, and whales, there is a layer of striated muscle (panniculus carnosus) underneath the subcutaneous fat layer (panniculus adiposus). The subcutaneous fascia resides underneath both the adipose and the panniculus carnosus muscle layers. This muscle layer is absent or minimally present in humans and in pigs. In these species, the subcutaneous fascia is directly underneath and continues within the subcutaneous adipose tissue without an intervening muscle layer (Figure 1). In humans, depending on the different anatomic locations, a single or several layers of superficial fascial bands can be found within the subcutaneous fat tissue [8,15]. It has been described that in abdominal and pelvic subcutaneous tissues, the superficial fascia separates the abdominal adipose tissue into superficial adipose tissue and deep adipose tissue [8,16]. Superficial fascia in healthy juvenile rodents has minimal adipocytes and is easily separable from deep fascia of the skeletal muscle [13,17], whereas human subcutaneous fascia tightly interdigitates subcutaneous fat. When superficial fascia splits into two sublayers, fat lobules are found enveloped inside the superficial fascia [8,15]. It has been shown that in rat hind limbs, adipose cells have a subcutaneous fascial origin. Adipose precursor cells are presumed to be derived from fascial cells, expressing markers similar to subcutaneous adipose-derived stem cells, and undergo spontaneous adipogenic differentiation in vivo during development [18]. However, a formal proof of a cell lineage relationship between fascial cells and adipocytes has not been performed.

Subcutaneous fascia also provides a structural scaffold for subcutaneous vascular and nerve networks. Fascia splits into two sublayers to envelop larger long vessels that are parallel to the skin surface and connects the vessel walls by attaching to their adventitia [8]. The vertical smaller vessels follow the fascial extensions (retinacula) to cross the subcutaneous fat and dermal connective tissue layers. The retinacula provide protection to these smaller vessels and prevent vessel displacement when the skin is stretched [8]. Similarly, lymphatic vessels also cross the subcutaneous tissue along the retinacula and are often completely enveloped by the fibrous septa that provide good support to their thin walls. The subcutaneous fascia has a high density of nerve endings belonging to the autonomic sympathetic system [19], where it provides a pathway for long tracts of larger nerves and protects the larger nerves from excessive stretching.

## 3. Composition of Subcutaneous Fascia

The functional properties of fascia come from its various stromal cells that are embedded in a gelatinous extracellular matrix rich in glycosaminoglycans, proteoglycans, polysaccharides, and high in water content [5,8]. Such a composition makes subcutaneous fascia highly deformable. The number of layers of the subcutaneous fascia and the amount of extracellular matrix they contain depend on age, gender, species, and anatomic locations [13,20].

The stromal cell composition of the fascia includes fibroblasts, pericytes, and adipocytes, which represent the cellular foundation of the subcutaneous fascia. Cohabiting with these stromal populations are immune cells such as resident dendritic cells, macrophages, mast cells, and lymphocytes [21].

Subcutaneous fascia constantly changes its length in response to stretch and compression. Here, fascial fibroblasts play a key role in managing such tension by rapidly remodeling their cytoskeleton without activating a fibrogenic program or turning into myofibroblasts [7]. Such homeostatic cytoskeletal responses appear to be specific to fascial tissue and do not occur in more densely packed dermal fibroblasts [12]. Fascial fibroblasts are positive for vimentin, fibroblast-specific protein 1 (FSP1; S100A4), CD26, and Sca1 and are negative for monocytic marker CD68, demonstrating their fibroblastic nature [22,23]. Recent single-cell RNA-sequencing studies have revealed two additional fascial fibroblast markers: *GPX3* (encoding Glutathione peroxidase 3) [24,25] and *MSX1* (encoding Msh homeobox 1) [26] that mark the fibroblasts of mouse subcutaneous fascia. Fascial fibroblasts further express specific sets of integrin complexes such as integrin α5β1 and αvβ3, which allow them to be connected to the extracellular matrix and sense the surrounding mechanical forces. The integrin-mediated intracellular signaling in fascial fibroblasts enables the fascia to adapt its mechanical responses with metabolic behaviors to ensure physiologic functions [27].

Physiological functions of subcutaneous fascia is ensured by additional tasks taken up by its resident fibroblasts including: (1) balancing extracellular matrix production and its modification by modulating matrix metalloproteinases (MMPs) and their inhibitors—tissue inhibitors of metalloproteinases (TIMPs) [3]; (2) maintaining stromal cell homeostasis by releasing growth factors such as connective tissue growth factor (CTGF/CCN2), transforming growth factor-beta (TGF-β), and fibroblast growth factor (FGF) [8]; (3) controlling inflammatory responses by interacting with and providing a niche for recruited immune cells [28]; and (4) maintaining balanced interstitial fluid pressure by regulating fluid pressure and flow that permeate through the fascia. When the fascial fibroblast undergoes a strain, the water inside is expelled toward the extracellular matrix. As soon as physical extension is complete, the fibroblasts return to their original size and re-establish contact with the extracellular matrix through the integrins, reabsorbing the water [7].

The origin and functional diversities of fibroblasts attract increasing attention in the field (reviewed in [29,30,31]). However, the heterogeneity of fascial fibroblasts is relatively understudied. A more recent study into the heterogeneity of fibroblasts within human deep fascia has identified at least two morphologically distinct fibroblastic types (reviewed in [32]). The transcriptomic profile and the exact physiological functions of different fibroblastic subsets in fascia requires further investigation using techniques such as single cell-RNA sequencing and genetic lineage tracing.

Subcutaneous fascia is composed of loosely packed interwoven fibers, unlike the fiber bundles of the dermis, and is rich with various collagen fibers of types I, III, IV, V, VI, XI, XII, XIV, and XXI. This amalgam of collagen types forms a unique structure that provides compliance [2] and resistance to tension and stretch, which commonly occurs in fascial tissues. As stated above, subcutaneous fascia has a unique orientation and architecture of collagen fibers that is vastly different from that found in dermis, tendons, ligaments, or aponeurotic sheets. The subcutaneous fascia also includes elastin fibers [2], which makes the young healthy fascia elastic. With age, the fascia and their retinacula extensions lose their elasticity however [8].

Subcutaneous fascia is rich in glycosaminoglycans, with a prevalence of hyaluronic acid, which plays a key role in providing hydration and viscosity to the fascial tissue, since it has the unique capacity of binding large quantities of water [7,33,34]. Extracellular hyaluronic acid is synthesized predominantly by fascial fibroblasts, as evidenced by Alcian blue staining, anti-hyaluronic acid binding protein (HABP) immunohistochemistry, transmission electron microscopy, and the expression of hyaluronan synthase 2 (HAS2) [20,22,35]. Fascial hyaluronic acid levels remain constant throughout life [35]. This contrasts with the dermis, where hyaluronic acid decline with aging, associated with skin dryness and reduced scarring [33]. Such a difference implies that a specific level of hyaluronic acid is required for normal physiological functions of superficial fascia [36]. Significant changes in hyaluronic acid levels may indicate an underlying pathology. The molecular mechanism of how fascia tissue maintains a relative constant level of hyaluronic acid requires further investigation.

Approximately two thirds of the volume of fascial tissues is composed of water [37]. The viscoelastic nature of fascia can only be observed in hydrated tissue. In healthy fascia, a large percentage of water is arranged in liquid crystals [38]. This is an ordered and structured form of H_3_O_2_^−^ water molecule at the hydrophilic surface. During massage, subcutaneous fascia absorbs physical pressure, and that pressure has been shown to build up the liquid crystalline water that is necessary for healthy physiological function [38].

## 4. Wound Repair by Subcutaneous Fascia

Traditionally, the wound healing process has been divided into four distinct but overlapping stages, namely hemostasis, inflammation, proliferation, and remodeling [39]. Wound repair has hitherto been thought to occur when the wound provisional matrix is formed as blood clots during hemostasis. In this theory, after the infiltration of inflammatory cells, the collagen-based extracellular matrix starts to rebuild the new tissue during the proliferation stage of fibroblasts. It was posited that the dermal fibroblasts from adjacent skin migrate into the fibrin-based provisional matrix, proliferate, and differentiate into myofibroblasts to form granulation tissue. Myofibroblasts express contractile alpha smooth muscle actin (α-SMA) that contracts and closes wounds [40]. Other series of experiments had indicated that granulation tissue was not necessary for wound contraction and closure by showing that wound closure was not impaired after repeated removal of the granulation tissue from wounds [41,42,43]. Instead, the excision of the wound edge caused immediate unwinding of skin contraction [42,44], suggesting that the contraction occurs from outside (wound edges), but not from inside (granulation tissue). Furthermore, studies by Harris and colleagues showed that the traction force of individual fibroblasts in wounds was significantly more than the force needed for cell movement and hypothesized that the extra force was used for dragging matrix fibers [45]. The recent findings of the pivotal role for subcutaneous fascia support these earlier findings [29,46].

The subcutaneous fascia responds to deep skin injuries by physically mobilizing into wounds, where it establishes a provisional wound scar [23]. The mobilized fascia tissue contains not only extracellular matrix, but also fascial fibroblasts, embedded blood vessels, macrophages, and peripheral nerves, which are needed for the initial wound repair. Fascial matrix serves as the provisional matrix in the wound bed, to accommodate and attract inflammatory cells from blood. By contrast, the dermal matrix remains immobile and refrains from physically mobilizing into wounds. This mobilization of a connective tissue assemblage is therefore unique to fascial connective tissue.

**Figure 2:**
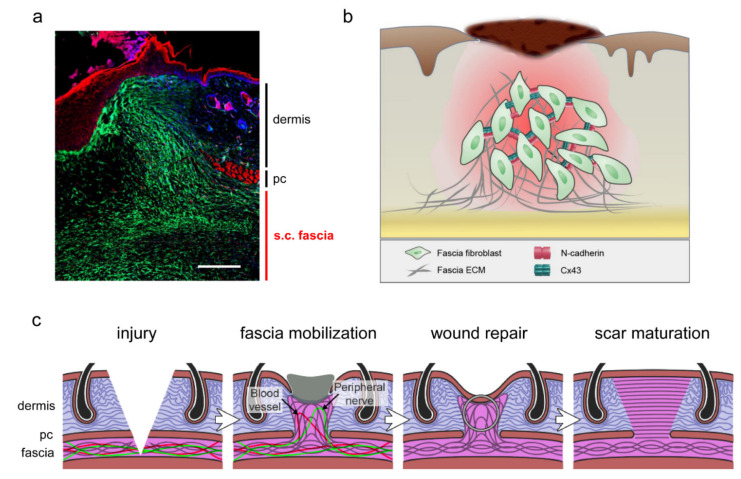
Wound repair by subcutaneous fascia. (**a**) Fluorescence microscopic image of a full-thickness excisional wound on the back of *En1*^Cre^; *R26*^mTmG^ transgenic mouse at day 7 post-wounding, showing that scar-forming fibroblasts from the subcutaneous fascia are mobilized into wounds. pc, panniculus carnosus; s.c., subcutaneous. Scale bars: 500 µm. (**b**) A scheme of fascial mobilization driven by collective migration of fascial fibroblasts via upregulation of N-cadherin and Connexin 43. (**c**) Scheme depicting the stages of wound repair by fascial mobilization. Fascial matrix is mobilized by resident fibroblasts to plug wounds with provisional matrix, initiate wound repair, and establish mature scars. (**b**) is adapted from a schematic figure in Jiang and Rinkevich, 2021 [29] with modifications. (**c**) is extensively modified from a schematic figure in Correa-Gallegos et al., 2019 [23].

Using genetic lineage tracing, anatomical fate mapping, and live imaging, we found that fascia-resident fibroblasts orchestrate the mobility of the connective tissue into wounds. Fascial mobility is directed by a single fibroblastic cell lineage termed Engrailed-1 lineage positive fibroblasts (EPFs), which is the profibrotic lineage fibroblasts responsible for skin scarring [47,48,49]. This specialized fibroblast type physically drags the fascia into the wound bed and subsequently into the skin surface in mice (Figure 2a). Genetically depleting fascial EPFs was performed by crossing the transgenic mice expressing Cre recombinase under the En1 promoter with the inducible diphtheria toxin receptor (DTR) transgenic mice, thereby restricting DTR to EPFs. Local delivery of diphtheria toxin into the subcutaneous fascia resulted in depletion of fascial EPFs. This genetic ablation strategy of fascial fibroblasts abolished the incorporation of fascial matrix into wounds and resulted in delayed wound healing. Furthermore, a porous film placed beneath the skin to prevent fascial fibroblasts from migrating upwards led to chronic open wounds that failed to close [23].

Our follow-up studies further support Harris’s fibroblast traction theory. We found that in response to injury, the expression of N-cadherin adherens-junctions [50] and Connexin 43 gap-junctions [51] were elevated specifically in fascial EPFs. These junctional structures transduce mechanical information, small molecules, and electrical activity, such as Ca^2+^ oscillation signals [52,53]. The adhesion and communication between fascial EPFs resulted in coordinated collective cell migration of fascial EPFs from wound edges toward the wound epicenter (Figure 2b). Therefore, fascial EPFs act as conveyor belts for dragging fascial matrix and plugging wounds.

Additional proof of the beneficial role for fascia in wound repair derives from its clinical use in surgery. For instance, aponeurotic fascia (deep fascia of the trunk) and fascia lata (deep fascia of the thigh) are often used as a surgical patch by plastic surgeons [54]. In a recent study, Yang and colleagues showed a method combining a superficial temporal fascia-free flap with thin split-skin grafting that was effective in promoting early wound closure in extensively burned patients with deep tissue defects in the posterior talocrural region. In all eleven patients, the fascial flaps survived, and wounds were repaired completely [55]. Fascial flaps have also been used as an additional treatment of chronic venous ulcers. A fascial pedunculated flap transferred from the sural area to the perimalleolar area before a free skin graft in the treated area significantly shortened healing time compared to the conservative therapy [56].

Several clinical practices target fascia to support wound repair and regeneration. For example, fascial tensile reduction sutures are used after surgical removal of keloids. The suturing of fascia in addition to the more superficial dermal sutures significantly relieves tension at the edges of the wound. This procedure also decreases the inflammation of the skin and prevents the re-formation of pathological scars after surgery [57,58].

Manipulation of the fascial system by compressing and stretching the subcutaneous fascia is commonly used by physiotherapists to resolve inflammation and manage pain. In modern exercise training, fascia stretching practices are becoming as important as aerobics and strength training. Fascia training helps to restore fiber distribution, orientation and alignment, and optimal tissue hydration and resilience. Simple manual pressure has been shown to cause alterations in the viscoelasticity of fascial tissue via deformation [2]. In a carrageenan-induced fascia inflammation model in mice, myofascial practices reduced neutrophil counts and increased levels of interleukin (IL)-4 and TGF-β, indicating an anti-inflammatory effect by modulating tissue biomechanics [59].

Acupuncture, a key component of traditional Chinese medicine, has been practiced to accelerate healing of chronic wounds [60] and to treat pathological scars to improve scar quality and reduce symptoms of pain and pruritus [61,62]. It has been demonstrated by ultrasonic elastomyography that the insertion and twisting of the acupuncture needles cause displacement of the subcutaneous fascia [63]. Such physical changes modulate the activity of fascial fibroblasts [64], which provide scientific evidence for the benefits of acupuncture to tissue repair.

Based on the observations that (1) depleting subcutaneous fascial fibroblasts or physically blocking subcutaneous fascial mobilization by placing a film beneath the skin resulted in chronic open wounds that fail to heal [23], and (2) the subcutaneous fascia becomes thinner with age [65], while the aged population is associated with a significant delay of wound healing and is subjected to a higher risk of developing nonhealing chronic wounds [66,67], we hypothesize that the key to understanding and clinically resolving non-healing wounds lies in modulating fascia mobility. However, at the moment, there is scarce clinical evidence that directly connects defects in subcutaneous fascia to chronic non-healing wounds, such as diabetic wounds, pressure ulcers, and venous leg ulcers, mainly because fascial research in wound healing is only at its infancy.

In healthy feet, the plantar fascia facilitates load sharing of the digits and reduce plantar pressure, whereas diabetic foot ulcers are often associated with abnormalities in plantar fascia, which severely compromise the capacity of fascial tissue to reduce focal plantar pressure [68,69]. Excessive pressure on fascia leads to a higher incidence of ulcer recurrence, since selective plantar fascia release has been shown to be effective in preventing and management of diabetic foot ulcers [70]. Further investigation of subcutaneous fascia in diabetic mouse models are expected to shed light on the cellular and molecular mechanisms underlying how defects in fascia impede chronic skin wounds from healing.

Necrotizing fasciitis is another typical example showing the involvement of fascia in skin wounding. These skin wounds and blistering are directly caused by bacterial infection of the subcutaneous and deep fascia. Necrotizing fasciitis is caused by one or more bacteria, with up to a third of cases involving methicillin-resistant *Staphylococcus aureus* (MRSA) [71]. Necrotizing fasciitis can start from a relatively minor injury, such as a small cut scratch on the skin, but becomes worse quickly with swelling and redness in the affected area and develops into fluid-filled blisters. If left untreated, the infection can spread quickly through the body and can be life threatening [72].

## 5. Fibrotic Outcomes of Fascia Repair

In mammals including humans, wound closure and scarring are two affixed outcomes of wound repair. The subcutaneous fascia functions not only as a prefabricated ready-to-use wound patch, but also as a repository of mobile scar tissue that closes wounds while simultaneously furnishing scar tissue.

We have found that the collective migration of fascial fibroblasts enables the fascial matrix to be mobilized into open wounds, and subsequently the mobilized fascial matrix undergoes substantial modifications into mature scars in wounds [50,51]. This process may be mediated by fascial matrix losing its elastic and hydrated properties and becoming a rigid fibrotic scar (Figure 2c). By contrast, wounds in oral mucosa and genital skin, where subcutaneous fascia is absent, heal with minimal scars [50,73]. The “dartos fascia” beneath genital skin has a striated carnosus muscle origin, and is not bona fide fascial tissue, further affirming that skin scarring is connected with the abundance and distribution of fascial tissue. Moreover, in the African spiny mice (genus *Acomys*), skin injuries are healed by scarless regeneration of the dermis, epidermis, and skin appendages including hairs, arrector pili muscles, sebaceous glands, adipocytes, and panniculus carnosus muscles [74,75]. Subcutaneous fascial tissue in the *Acomys* back-skin is remarkably thinner than that of the laboratory mice (*Mus musculus*) [74]. *Acomys* back-skin also exhibits lower skin biomechanics and wound tissue stiffness [76,77] as compared to the laboratory mice, which is equitable with its diminished fascial tissue. The above indicate that the abundance of the skin’s subcutaneous fascia may explain the diversity seen in the skin’s wound responses to injury, namely scarring or regeneration, across anatomic skin location and species.

The arrangement and thickness of subcutaneous fascia also vary with age and gender. The subcutaneous fascia becomes thinner during aging [65], consistent with aged populations being at higher risk of developing non-healing wounds and having lower risk of extreme scarring [66,78]. Superficial fascia is constantly thicker in women compared to men at various anatomical sites. For instance, female subcutaneous fascia at back is 15% thicker than male (0.16 mm vs. 0.14 mm) [13], while back skin in women is 55% thinner than in men (1.5 mm vs. 2.3 mm) [46]. The combination thicker subcutaneous fascia and thinner skin implies that injury of female skin more easily breaches the fascial compartment. This will mobilize matrix more often in women and inflict more pathological scars. This is the case with hypertrophic scars and contractures that are more prevalent in women [78].

Therefore, fascia-specific strategies may be a promising novel therapeutic landscape on which to prevent scars and enhance wound repair. One possible strategy will be to target fascial fibroblasts, which display molecular and functional features that are different from papillary and reticular dermal fibroblasts [29,46]. Fascial fibroblasts possess higher potency in proliferation and in collagen lattice contraction in vivo and hence are profibrotic and more active than dermal fibroblasts [79]. Fascial fibroblasts resemble features of fibroblasts derived from hypertrophic scars and keloids, including high expression of NOV (nephroblastoma overexpressed, or CCN3), CD26 (DPP4), and FAP (fibroblast activating protein) [23] to produce more of the proteoglycan versican [80] but less collagenase (MMP1) [81]. This strategy is supported by the experimental evidences that depletion of fascial cells [23] or fascial EPFs [50] by genetic ablation remarkably reduced scar sizes after full-thickness excisional wounds in juvenile mice.

The second strategy is targeting fascial movement. Since a complete blockage of subcutaneous fascial mobilization results in chronic wounds [23], it will not be a therapeutic option for scarless repair. Nevertheless, partial inhibition of fascial mobilization by targeting N-cadherin or Connexin 43 with chemical inhibitors or genetic ablation in fascial EPFs have shown promising effects on reducing scarring in mice [29,50,51]. These treatments significantly reduce the amount of fascial matrix in wounds, resulting in reduced fascia-derived collagen content and infiltrated macrophages, and leading to substantially reduced scar size.

The third strategy is to prevent the undesired fibrotic modification of fascial matrix after its mobilization in wounds. However, thus far, little is known about the biochemical processes involved in fascial matrix fiber cross-linking, maturation, and degradation in the wound microenvironment. The matured scar matrix contains less elastin fibers than the fascial matrix, which makes scar tissue rigid [82]. The increased matrix stiffness and increased tissue strain promote excessive scar formation [83]. Further investigation will be required to establish whether the degradation of elastic fibers is one of the matrix modifications after fascial mobilization.

Whereas humans and mammals employ fascia primarily for scarring and tissue contraction, in lower vertebrates, fascial tissue is crucial for tissue regeneration after wound repair. For instance, in *Xenopus laevis* froglets, subcutaneous fascial cells have been shown to contribute to scarless skin regeneration. By performing transplantation and cell-fate tracing using chimeric froglets that have GFP-negative skin and GFP-labeled subcutaneous fascial tissues, Otsuka-Yamaguchi and colleagues found that subcutaneous fascial cells became blastema-like cells with activated *Prrx1* limb enhancer, and contributed to regenerating the skin, especially the dermis, after an excision injury [84]. Recently, Prrx1^+^ fibroblasts have been shown to be responsible for scar formation of the ventral skin in mice [85], but a determinant of a successful limb regeneration in *Xenopus laevis* [86]. Therefore, understanding the key similarities and differences in fascial fibroblasts between mammals and lower vertebrates may provide molecular and cellular insights into scarless wound healing.

## 6. Implication of Fascia in Fibrotic Diseases

Conditions that alter subcutaneous fascia such as trauma, surgery, diabetes, and aging [87], expose patients to a higher risk of developing wound healing alterations.

Hypertrophic scars and keloids are a typical example. Tension is a factor predisposing the onset of pathological scars. Injury or abnormalities of subcutaneous fascia alters the dynamics distribution of tissue tension. In consequence, the peripheral tension at the margins is considerably elevated. In keloids, the major recorded force lines are found outside of the scar and result in constant pulling and expansion of the tissue [88,89]. Furthermore, subcutaneous fascia has a high density of sympathetic nerve endings. In response to the stimuli of anomalous tension upon injuries, nerve endings in subcutaneous fascia release large amounts of neuropeptides such as substance P, neurotrophin 3, and neurotrophin 4 [3]. This process provokes a release of neuropeptides from other cell types including fascia fibroblasts, triggering a neuroinflammatory reflex arc [3,88,90]. This evidence well explains the clinical observation that an increase of nerves and an accumulation of neuropeptides are found in hypertrophic scars and keloids [89].

In addition, the fascial tissue itself can undergo fibrosis during pathologic conditions, such as in Dupuytren’s disease [91] and in eosinophilic fasciitis [92]. Dupuytren’s disease is characterized by fibrosis of the fascia underlying the skin of the palm and fingers [91]. The etiology of Dupuytren’s contractures has been unclear until a recent scRNA-seq study uncovered a unique fibroblast subset expressing intercellular adhesion molecule 1 (ICAM1) isolated from Dupuytren’s nodules. The ICAM1^+^ fibroblasts secrete high levels of IL-6 and IL-8 and exhibit a direct chemotactic activity. Fascial fibroblasts in Dupuytren’s nodules are key drivers of inflammation, whose feedback further sustains fibroblastic activation and fibrosis [93].

Eosinophilic fasciitis also features with dense fibrosis, marked thickening and inflammation of subcutaneous fascia and deep fascia in the epicenter of the lesion. Fibrosis can extend from the fascia to the dermis, eventually resulting in painful swelling, erythema, and progressive contracture of skin at affected areas [94]. The fascia also accumulates eosinophils, lymphocytes, and other plasma cells [95]. Eosinophilic fasciitis is thought to be caused by abnormal allergic or inflammatory reaction, including elevated serum IL-5, but the exact etiology is unknown. Recently, it was shown that the fascial fibroblasts of affected areas have increased gene expression of *TGFB1* and *CCN2* (*CTGF*), which is correlated with higher expression of type I collagen and fibronectin and increased production of TIMP-1, an inhibitor MMP-1 (collagenase) [95].

These examples clearly demonstrate that fascial abnormalities contribute to pathological fibrosis. Multiple factors within fascial tissue are involved in fibrotic processes, such as defects in tension loading, dysregulated release of neuropeptides, inflammatory mediators, growth factors, and matrix modifying enzymes.

## 7. Perspective in Organ Fibrosis

The fascial system consists of sensing tension and load. Subcutaneous fascia is connected to and a part of the fascial system. It has been shown that the local injury that changes the tension in subcutaneous fascia could spread the fibrotic responses by activating quiescent fibroblasts via mechanotransduction in otherwise remote locations [96] and potentially affect the health status of the visceral organs and the entire body. Many chronic conditions, such as heart failure and chronic obstructive pulmonary disease (COPD), often show fibrotic alterations in the fascial system.

Similar to the subcutaneous fascia underlying the skin, the loose connective tissues directly beneath the mesothelium of the viscera are defined as visceral fascia [97]. Visceral fascia surrounds organs in cavities, including liver, heart, lung, kidney, esophagus, parietal peritoneum, and parietal pleura. The healing process undergone by the internal organs, for example, after surgery, is the same as that observed for the skin. There are a variety of organ systems that demonstrate chronic pathologic fibrotic response to injury and always associate with alterations in visceral fascia, such as heart failure following ischemic insult, COPD, and idiopathic pulmonary fibrosis. One remarkable example is the surgical adhesions, which are the result of lack of sliding between the various fascial layers. This absence or reduction of movement causes an inflammatory environment, which creates adhesions [98]. Recent studies have clearly demonstrated that surgical adhesions are cicatricial events driven by mesothelium and the associated visceral fascia [99,100,101]. Therefore, it is plausible that there is a universal mechanism underlying the fascia associated fibrotic event. Dissection of molecular details in future investigations of fascial system will provide a promising therapeutic target to prevent, ameliorate, and treat skin scarring and organ fibrosis.

## 8. Concluding Remarks

The discovery of subcutaneous fascial mobilization signifies a newly found and pivotal role for fascia in wound repair and regeneration, with two critical factors, collective migration of fascial fibroblasts, and mobilization of fascial matrix. The in vivo experimental in mice provide cellular and molecular basis supporting Gross’s model that wound contraction and healing is driven by fascial fibroblast migration from edges of wounds toward wound centers, and Harris’s model wherein fascial fibroblasts exert traction on the fascial connective tissue to enact mobilization into wounds. The recent findings that fascia is a major source of wound provisional matrix and of scars rather than de novo produced matrix, reconfigure our traditional view of wound repair. This new wound pathomechanism offers a novel therapeutic space to curtail pathological wound repair and fibrotic responses and induce scarless regenerative healing across a range of medical settings.
